# Pharmacogenetic testing for thiopurine drugs in Brazilian acute lymphoblastic leukemia patients

**DOI:** 10.1016/j.clinsp.2023.100214

**Published:** 2023-05-06

**Authors:** Guilherme Suarez-Kurtz, Cristina Wiggers Almeida, Eduardo Chapchap, Márcia Trindade Schramm, Maura Rosane Valério Ikoma-Coltutato, Mecneide Mendes Lins, Teresa Cristina Cardoso Fonseca, Thais Ferraz Aguiar, Mariana Emerenciano

**Affiliations:** a Divisão de Pesquisa Clínica e Desenvolvimento Tecnológico, Instituto Nacional de Câncer, Rio de Janeiro, RJ, Brazil; b Hospital Federal da Lagoa (HFL), Rio de Janeiro, RJ, Brazil; c Hospital Universitário Pedro Ernesto, Universidade do Estado do Rio de Janeiro (UERJ), Rio de Janeiro, RJ, Brazil; d Hospital Albert Einstein, São Paulo, SP, Brazil; e Hospital do Câncer I, Instituto Nacional de Câncer, Rio de Janeiro, RJ, Brazil; f Prontobaby Hospital da Criança Ltda, Rio de Janeiro, RJ, Brazil; g Fundação Amaral Carvalho, Jaú, SP, Brazil; h Sabin Medicina Diagnóstica, Brasilia, DF, Brazil; i Instituto de Medicina Integral Professor Fernando Figueira, Recife, PE, Brazil; j Hospital Manoel Novaes-Santa Casa de Misericórdia de Itabuna, Itabuna, BA, Brazil; k Instituto Estadual de Hematologia Arthur Siqueira Cavalcanti, Rio de Janeiro, RJ, Brazil; l Divisão de Pesquisa Básica e Experimental, Instituto Nacional de Câncer Rio de Janeiro, RJ, Brazil

In a review article for Clinics in 2018,^1^ one of us presented a personal perspective of pharmacogenetic (PGx) testing in oncology in Brazil, highlighting the drug-gene pairs for which there are international consensus guidelines for PGx-informed drug prescription. In 2019, PGx testing for irinotecan:UGT1A1, fluoropyrimidines:DPYD and thiopurines:TPMT/NUDT15 was implemented at Instituto Nacional de Câncer (INCA) in Rio de Janeiro.^2^ Here, the authors present results for thiopurines:TPMT/NUDT15, to highlight the development and current status of INCA´s PGx program.

PGx testing for thiopurines was designed as an extension of an ongoing project^3^ focused on genomic deletions in Acute Lymphoblastic Leukemia (ALL) patients from INCA and nine other oncology centers in Brazil. The study was approved by INCA´s Ethics Committee (CAAE 33709814.7.1001.5274), routine procedures for receiving, processing, and storing DNA samples were established and validated allele discrimination TaqMan assays were applied to selected TPMT and NUDT15 single nucleotide polymorphisms (SNPs; Supplementary Table 1). Selection of the target SNPs, assignment of TPMT, NUDT15, and compound (combined TPMT and NUDT15) metabolic phenotypes according to the TPMT and NUDT15 genotypes, and recommendations for initial thiopurine dosing followed the Clinical Pharmacogenetics Implementation Consortium (CPIC) updated thiopurine guideline.^4^

From the inception of the PGx program until now, 430 ALL patients were successfully genotyped for TPMT and NUDT15 SNPs (Supplementary Table 2). The frequency distributions of TPMT and NUDT15 alleles, genotypes and diplotypes, and assigned metabolic phenotypes are shown in Table 1. Minor Allele Frequency (MAF) ranged between 0.012 (NUDT15 rs116855232) and 0.049 (TPMT rs1142345). There were no significant deviations from Hardy-Weinberg equilibrium at any locus, such that homozygosis for the wildtype alleles ranged from 0.907 (TPMT rs1142345) to 0.977 (NUDT15 rs116855232), and heterozygosis varied between 0.023 (TPMT rs116855232) and 0.088 (TPMT rs1142345). Homozygosis for the variant (minor) allele was detected only at the TPMT rs1142345 locus. The allele and genotype frequency of the interrogated SNPs in both TPMT and NUDT15 are in excellent agreement with data from Southeast Brazilian cohorts.^5^^,^^6^

**Table 1:** TPMT and NUDT15 polymorphisms in Brazilian ALL patients.

Gene / SNPs	Genotype frequency								MAF	
TPMT	wt/wt			wt/var			var/var			
rs1142345	0.907			0.088			0.005		0.049	
rs1800462	0.951			0.049			0.000		0.024	
rs1800460	0.972			0.028			0.000		0.014	
NUDT15										
rs116855232	0.977			0.023			0.000		0.012	

**Gene**	**Diplotype frequency (%)**									

TPMT	*1/*1	*1/*2	*1/*3A		*1/*3C	*2/*3ª		*2/*3C	*3ª/*3C	*3C/*3C
	88.4	2.3	4.0		4.4	0.2		0.2	0.2	0.2

**Enzyme**	**Assigned metabolic phenotypes (%)**									

	Normal			Intermediate			Poor		Compound	
	(NM)			(IM)			(PM)		Intermediate	
TPMT	88.4			10.7			0.9		‒	
NUDT15	97.7			2.3			0.0		‒	
Compound	86.5			11.6			0.9		0.9	

wt, Wild-Type allele; var, Variant allele; MAF, Minor Allele Frequency.

The Normal Metabolizer phenotype (NM) was assigned to the vast majority of patients for TPMT (88.4%) and for NUDT15 (97.7%), whereas the Intermediate Phenotype (IM) was assigned to 10.7% (TPMT) and 2.3% (NUDT15) patients. Poor metabolizers (PM) were rare (TPMT, 0.9%) or absent (NUDT15). Four patients (0.9%) were heterozygous carriers of both NUDT15 rs116855232 and TPMT no-function alleles and consequently were assigned the compound IM phenotype, which is associated with lower thiopurine tolerance compared with IMs for either TPMT or NUDT15.^4^

Based on the individual TPMT and NUDT15 combined metabolic phenotypes, four distinct recommendations for thiopurine initial dosing were made (Fig. 1): i) Start treatment with the usual thiopurine dose: patients with the NM phenotype for both enzymes (86.5%); ii) Consider starting the treatment with reduced thiopurine doses: IMs of either TPMT or NUDT15 (11.6%); iii) Drastic reduction of the initial dose and reduced frequency of administration: PMs: of either or both enzymes (0.9%); iv) Consider a large reduction of the initial thiopurine dosing: compound IMs (i.e. IM for both enzymes) in view of the potential highly increased risk of thiopurine toxicity.

**Fig. 1:**
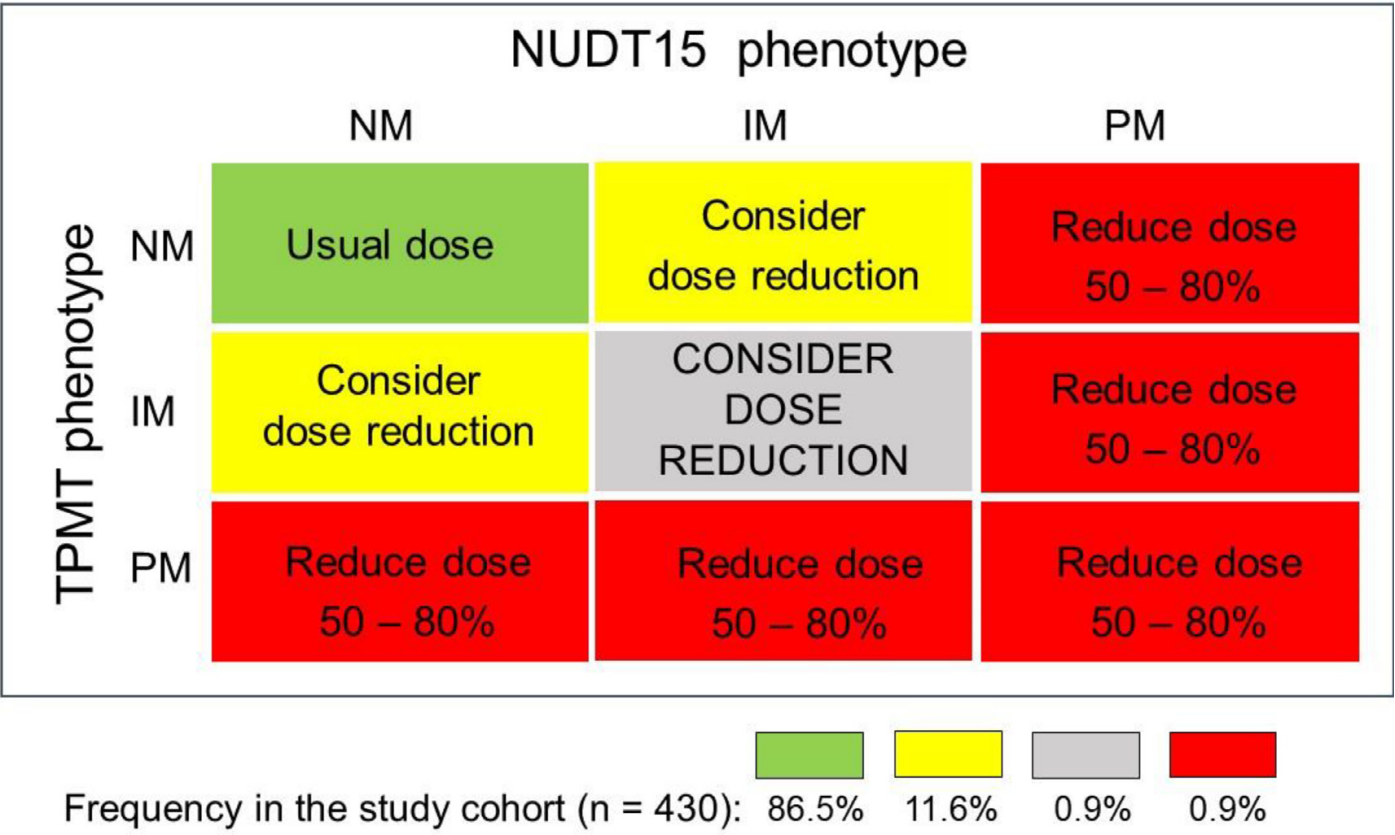
Diagrammatic plot of mercaptopurine dosing recommendation for 430 ALL patients, based on the individual compound metabolic phenotypes for TMPT and NUDT15 enzymes. The recommendations follow the updated CPIC guideline.^4^ The proportion of patients receiving each recommendation is shown at the bottom.

The individual TMPT and NUDT15 genotypes and metabolic phenotypes, and the thiopurine dosing recommendation were included in a concise report, which was conveyed by institutional email to the Principal Investigator of each participating institution. The reports cautioned that the dosing recommendation applied to the initial thiopurine dose; subsequent doses should be adjusted based on the degree of myelosuppression and the disease-specific guidelines. The report contained the following disclosure statements: i) The dosing recommendations are based on the CPIC guidelines according to the polymorphisms genotyped; ii) The possibility of influence of other, not interrogated PGx variants cannot be excluded; iii) Adherence to the dosing recommendations is a decision of the prescribing physician.

PGx testing for thiopurines is a landmark in the adoption of PGx as a major instrument for precision (personalized) medicine, and as such led to the creation of the CPIC guidelines.^7^ The original thiopurine guideline contemplated only TMPT polymorphisms; the inclusion of NUDT15 polymorphisms in the 2018 updated guideline attests to the dynamics of PGx research but also highlights the challenge that dosing recommendations must be updated as novel evidence of PGx associations emerges. Implementation of PGx testing at INCA to inform the prescription of mercaptopurine to ALL patients is a pioneering initiative within the Brazilian Public Health System, which hopefully will prompt similar programs in other medical centers in Brazil, whether public or private. Financial support from the Brazilian agencies Decit/MS, FAPERJ, and CNPq, and the commitment of a dedicated task force at INCA were decisive factors for the success of the program and assured its continuity throughout the COVID-19 pandemic.

## Conflicts of interest

The authors declare no conflicts of interest.

## Appendix Supplementary materials

Image, application 1

## Footnotes

Supplementary material associated with this article can be found in the online version at doi:10.1016/j.clinsp.2023.100214.
