# Supplementary material for: Pharmacogenetic testing for thiopurine drugs in Brazilian acute lymphoblastic leukemia patients
Source: Clinics (Sao Paulo). 2023 May 6;78:100214. doi: 10.1016/j.clinsp.2023.100214 (PMC10193153; doi:10.1016/j.clinsp.2023.100214)
Supplement: Supplementary file 1 [file mmc1.docx]

CLINICS-D-23-00028_Supplementary Material

**Supplementary Table 1** Genetic polymorphisms in TPMT and NUDT15.

| **Gene rsID** | **Polymorphism** | **Trivial name** | **Enzymatic Activity** |
| --- | --- | --- | --- |
| **TPMT** |  |  |  |
| rs1142345 | c.719A>C | TPMT*3C (TPMT*3A)^a^ | Null |
| rs1800460 | c.460G>A | TPMT*3B (TPMT*3A)^a^ | Null |
| rs1800462 | c.238G>C | TPMT*2 | Null |
| **NUDT15** |  |  |  |
| rs116855232 | c.415C>T | NUDT15*2, NUDT15*3 | Null |

rsID, Identification Hugo gene nomenclature committee.

^a^ TPMT*3A comprises both c.719A>C and c.460G>A.

**Supplementary Table 2** Demography, clinical and diagnostic characterization of pediatric and adult ALL patients.

| **Pediatric ALL** | | **Adult ALL** | |
| --- | --- | --- | --- |
| **Variable** | **n (%)** | **Variable** | **n (%)** |
| Age (years) |  | Age (years) |  |
| <5 | 123 (35.8) | 18‒40 | 53 (61.6) |
| 5‒10 | 118 (34.3) | 41‒60 | 29 (33.7) |
| 11‒17 | 103 (29.9) | 61‒67 | 4 (4.7) |
| Sex |  | Sex |  |
| Female | 131 (38.1) | Female | 32 (37.2) |
| Male | 213 (61.9) | Male | 54 (62.8) |
| WBC count (×10^9^/L) |  | WBC count (×10^9^/L) |  |
| <50 | 260 (75.6) | <50 | 56 (65.1) |
| ≥50 | 84 (24.4) | ≥50 | 30 (34.9) |
| ALL Subtype* |  | ALL Subtype* |  |
| B-ALL | 295 (85.7) | B-ALL | 65 (75.6) |
| T-ALL | 49 (14.3) | T-ALL | 21 (24.4) |
| Total | 344 | Total | 86 |

ALL, Acute Lymphoblastic Leukemia; WBC, White Blood Cell Count.
